# Global sales and operations planning: A multinational manufacturing company perspective

**DOI:** 10.1371/journal.pone.0257572

**Published:** 2021-09-21

**Authors:** Marcelo Xavier Seeling, Tobias Kreuter, Luiz Felipe Scavarda, Antônio Márcio Tavares Thomé, Bernd Hellingrath

**Affiliations:** 1 Industrial Engineering Department, Pontifícia Universidade Católica do Rio de Janeiro, Rio de Janeiro, Brazil; 2 Chair for Information Systems and Supply Chain Management, Westfälische Wilhelms-Universität Münster, Münster, Germany; University of Salento, ITALY

## Abstract

The purpose of this paper is to analyse the global Sales and Operations Planning (S&OP) process and investigate the steps to support consolidated business planning in worldwide operations and large-scale supply chains. The paper conducts a case study at a multinational manufacturing company applying an abductive approach. It combines the deductive logic from theory and the inductive logic from field observation in an attempt to elaborate further on theory on global S&OP. The analysis is structured and guided by a novel framework for global S&OP, which is developed based on the theoretical background and the case study findings. The research findings characterise the S&OP process for global operations and identify challenges related to the need to synchronise the subsidiaries’ S&OP efforts worldwide to deal with different contingencies of these subsidiaries, and to manage and analyse a large amount of information gathered. The research reveals how the subsidiaries’ performance is analysed by top executives along the global S&OP process, feeding strategic initiatives in the organisation and identifying business opportunities like benchmarking among subsidiaries, synergies with other management practices, and global gains. This paper offers a novel investigation of the global steps on S&OP in a real-life setting, offering a well-documented characterisation of the process that goes beyond the traditional local approach. Moreover, it is the first study to reveal challenges and expected outcomes of such a global perspective for S&OP. The theoretical advancements of S&OP research offered herein aid scholars, opening avenues for middle-range theorising, highlighting the cross-disciplinary nature of the domain, and discussing the use of concepts from related disciplines like Economics, Psychology, and Information Systems. The research findings can also assist executives, especially from multinational manufacturers, in their efforts to consolidate global planning.

## Introduction

The fierce competition in the global markets has forced multinational manufacturing companies to review their strategies, systems, and processes to succeed [1]. Multiple environmental factors complicate today’s business management, including globalisation, an increasing supply chain complexity, and, just recently, the impacts of the worldwide COVID-19 pandemic [2]. To face turbulent markets and uncertain economic environments, global corporations are putting more focus on their supply chain processes [3, 4] and supply chain planning [5], in which sales and operations planning (S&OP) has become one critical factor [6]. S&OP is a management process leading to cross-functional coordination and integration [7, 8]. It is situated at the tactical level and is conducted periodically, usually once a month [9, 10]. S&OP balances demand and supply within the company and along the supply chain [11]. If properly implemented, S&OP may contribute to increasing the company’s supply chain and overall performance [12–14].

The interest in S&OP has raised significantly from both academics and practitioners in the last decades, with an increasing number of implementations in different industries worldwide [11, 15] and a research growth reflected in recent literature reviews on the subject (e.g., [15–18]). Nevertheless, there are still knowledge gaps and research opportunities embracing the lack of a complete characterisation of the S&OP process [19–22], including the global S&OP in multinational manufacturing companies [18, 20, 23, 24] and large-scale supply chains [25]. This is reflected in the scarcity of S&OP analyses beyond the standard local five-step approach to S&OP [26] and including additional steps in companies with global operations [20, 24], aiming to integrate different local S&OP within subsidiaries into a worldwide S&OP. Consequently, international or global S&OP processes should be further explored by case studies, as recently called for in Kreuter et al. [18] and in Stentoft et al. [27].

It is important to address this gap, as manufacturing companies with several subsidiaries in different countries and global supply chains may be required to go beyond the local five-step approach to reach a global S&OP, aiming for consolidation and integration of the different plans worldwide [28–31]. Therefore, there is a need to investigate how the steps of reaching a global S&OP are deployed in organisations with operations in different countries and large-scale supply chains, and what the results of such global perspective for S&OP are. This leads to the following research questions (RQs).

RQ1: How is the S&OP process performed for global operations in multinational companies?
RQ2: What are the challenges and results of the consolidation and integration of the different local S&OP worldwide?

The purpose of this research is to contribute to a better understanding of the global S&OP, characterise its process, and analyse the under-researched global steps to support consolidated business planning in worldwide operations and large-scale supply chains. This paper presents the findings from a case study performed in a multinational manufacturing company, including two Latin American subsidiaries (Brazil and Mexico) and its headquarters in the United States of America (USA). The paper contributes to theory elaboration and theory development through case study research [32, 33], synthesising the main research findings into a framework for global S&OP. It thereby addresses a recent call for more theory-informed empirical research in S&OP [18, 19]. The research findings are discussed from a cross-disciplinary view, opening an extended perspective on S&OP going beyond the traditional focus from Operations Management.

The remainder of the paper is structured as follows. Section 2 presents the theoretical background. Section 3 describes the research method. Section 4 provides the research findings on the local and global steps of the S&OP process, addressing RQ1. Section 5 analyses challenges and global results associated with the consolidation and integration of the different local S&OP worldwide, addressing RQ2. Section 6 offers a discussion on the theoretical advancements and practical implications of the research. In the final section, conclusions and suggestions for future research close the paper.

## Theoretical background

A successful S&OP implementation increases profit by decreasing costs and increasing revenues and improves operations’ performance regarding customer service, demand forecast, inventory management, and manufacturing resources [12, 13, 15]. However, successful implementations obtaining the full benefits are challenging and still rare [21, 22]. A central reason for this is the lack of a complete understanding of S&OP [34], which requires a wide-ranging characterisation of the process [19–22]. This section presents current literature findings aiming to address the issues regarding the S&OP understanding and process characterisation. The following two subsections offer the S&OP building blocks and the S&OP process steps from a local to a global scope. The findings are structured by two central frameworks identified in the S&OP literature.

### S&OP building blocks

**Fig 1:**
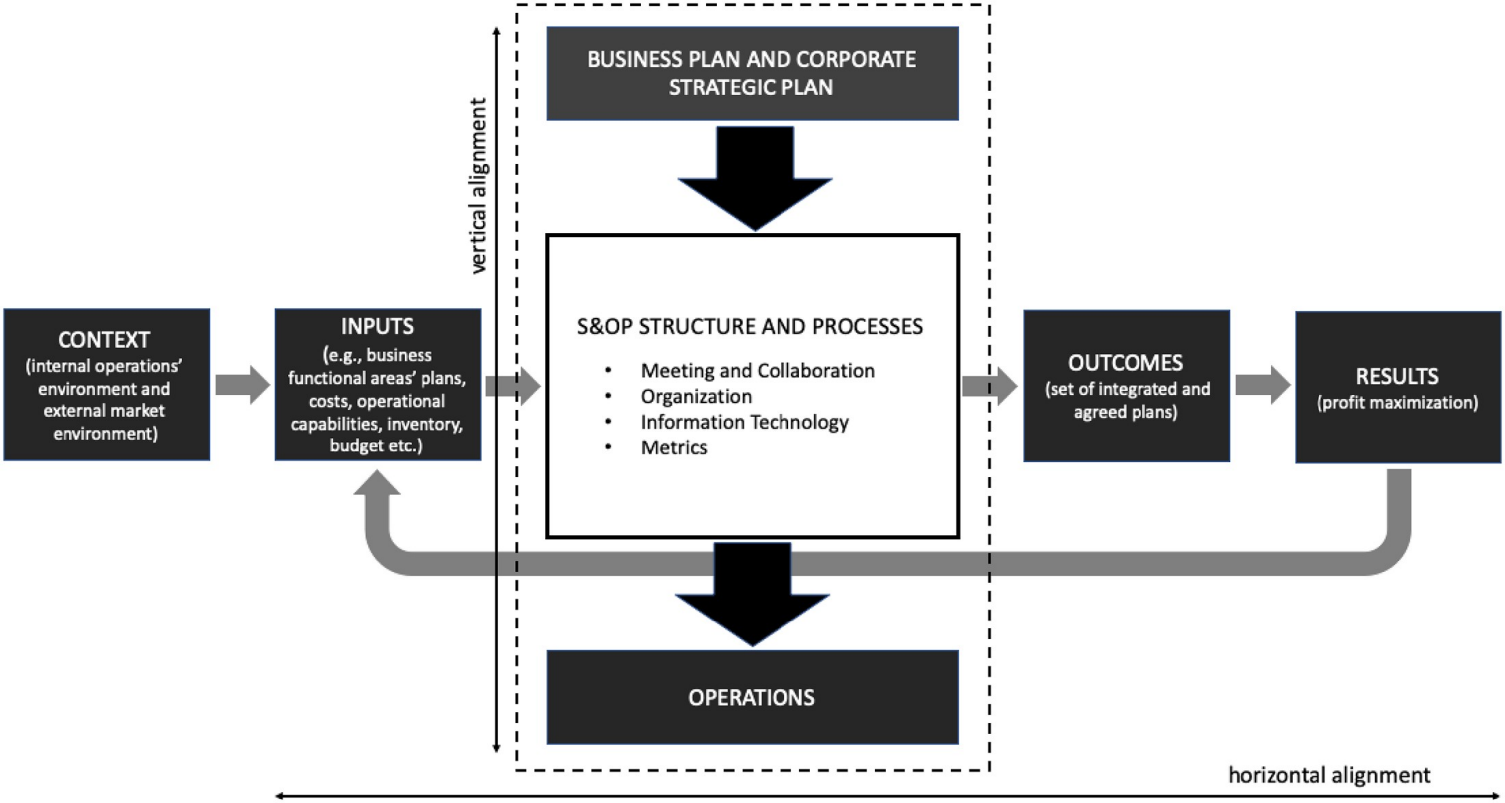
S&OP building blocks (Adapted from Thomé et al. [10]).

Based on a systematic literature review, Thomé et al. [10] offer an S&OP framework that contributes to an S&OP design understanding. It synthesises the main building blocks and design parameters of S&OP [35] and contemplates vertical and horizontal alignments systematically and holistically [36]. It is consistent with Kathuria et al. [37], where vertical alignment bridges strategy to operations, while horizontal alignment refers to cross-functional integration. Fig 1 depicts the S&OP framework with the main building blocks, which are described next.

The main building blocks of S&OP are *context*, *business and corporate plans*, *inputs*, *structure and processes*, *outcomes*, and *results*. *Context* refers to the enterprise’s operating environment. It embraces aspects such as geographic region, industry type, manufacturing strategy, product-process matrix, product aggregation level, hierarchical planning, and planning horizon [10]. The context-based nature of S&OP is relevant and has been highlighted in the literature (e.g., [16, 38, 39]), making it a relevant building block for the S&OP comprehension. The *Business Plan* of the framework is the company’s highest planning level, encompassing the corporate Strategic Plan [10]. *Inputs* to the S&OP process include functional business areas’ plans, forecasts, capacities, and constraints. The *outcome* is the integration of different plans into a single one, reconciling the plans horizontally from diverse functional areas and ensuring vertical alignment between the company’s strategic plan and operations [7, 40, 41]. The expected main *result* is profit optimisation [42].

*Structure and Processes* is the central building block, being the core of S&OP, transforming inputs into outputs. It is composed of four design parameters: meeting and collaboration, organisation, information technology (IT) and metrics [10, 35]. Meeting and collaboration evaluate the effectiveness of the human component in the S&OP process [42], including participants, collaboration and trust, and cycle meetings. Organisation comprises the structure to execute the process, including the establishment of a cross-functional team, agenda, and process steps. IT refers to software, interfaces, hardware, and communication systems used to support the process execution. Metrics covers all key performance indicators (KPIs) and measurement procedures used to monitor results and continuous improvements in the operations area and the overall business, as well as the S&OP dashboards, including measuring the process management itself and its effects on corporate effectiveness and efficiency [43]. S&OP effectiveness measures can be related to forecasting accuracy, resource adherence (e.g., capacity capability, inventory), trade-off measures (cross-functionality with demand plan vs production plan and alignment with strategy and reward system), plans adherence and actual vs target [41].

Thomé et al.´s [10] framework evolved from Grimson and Pyke´s [42] S&OP maturity model. It is considered an “integrative framework” [16], that describes S&OP as a whole and not as a partial process [36], being generalisable and not restricted to specific contexts [15]. Lately, the framework was adapted for the coordination mechanisms [7], other supply chain integration practices, such as CPFR [44], a multiple case study of the food industry in North Europe [35], metrics [41], process industries [15], teaching cases in Operations Management [21], a context-based S&OP framework [16], and recently in a case study in the chemical industry in South America [24].

### S&OP process steps: From local to global scope

On a local scale, the S&OP process is usually performed in a monthly planning cycle embracing the following five steps [26]: data gathering, demand planning, supply planning, pre-meeting, and executive meeting. These five steps are observed in a majority of S&OP studies (e.g., [11, 40, 42, 43]), and are discussed next.

Data Gathering (step one) occurs when the month results are closed [11] and the company’s information system (e.g., Enterprise Resource Planning—ERP) is fed with data from different functional business areas. Demand Planning (step two) is when sales and marketing teams review historical data and the sales forecast, include the impact of new product launches, advertisement and promotions and other trade initiatives to generate the unconstrained demand plan. Supply Planning (step three) is when the demand plan received from step two is simulated, including capacity constraints [42] and inventory figures [10]. Representatives from operations (e.g., production, logistics, research and development—R&D) work together in this step [10].

Pre-meeting (step four) is when representatives from different functional business areas (e.g., sales, marketing, and operations) discuss the gaps between demand needs and constrained supply capacities. They work together to develop mitigation plans to solve the problems [40] and prepare a financial review [31]. The agreed S&OP plan proposal with a financial analysis and the unresolved issues and trade-offs that need senior managers’ decisions are taken to the executive meeting (step five). Company’s senior managers and executives take part in step five: (i) to approve the pre-meeting S&OP plan or choose another course of action; (ii) to authorise changes in production rates or supply costs that go beyond S&OP team autonomy; (iii) to analyse and compare the financial S&OP plan vs Business Plan (budget) and to decide which adjustments are needed; (iv) to resolve pending issues without agreement from the Pre-meeting and any other relevant issues raised; and (v) to review KPIs, projects’ progress, product launches and to make decisions [26].

However, competition in global operations, supply chains and markets [4, 6] makes the implementation and execution of a single S&OP process for an entire multinational corporation unsuitable [34, 35]. As multinational manufacturing companies embrace several subsidiaries in different countries with large-scale supply chains, they require the consolidation and integration of the different S&OP plans worldwide [28–31]. Additionally, within their geographic growth, corporations face the so-called “fight over decision rights” among their headquarters with global functions and their subsidiaries with similar local functions in specific countries [31]. Thus, the S&OP process sometimes needs to be divided up according to certain criteria (e.g., region, country, business unit, and product line) that fits the company [20, 29] and then it has to be consolidated to: (i) harmonise the integration of plans worldwide [29]; (ii) ensure vertical alignment and allow global visibility [24]; and (iii) achieve the organisation’s global strategy [25, 29]. This consolidation is the extension from a local to a global S&OP [28]. Within this context, there may be two additional steps for the S&OP process in global corporations: global roll-up and global executive meeting [24, 26].

In the global roll-up (sixth step), S&OP information from subsidiaries or business units worldwide is consolidated. Organisation’s most senior executives as the chief executive officer (CEO), chief operations officer (COO), chief financial officer (CFO), other chief officers (C-suite), plus regional or divisional presidents, and global vice presidents meet at the global executive meeting (seventh step). They analyse the consolidated information, KPIs, financial results, global projects advancements, relevant issues and make decisions [26]. Within steps six and seven, the S&OP planning horizon is extended and tends to merge with the multi-year strategic plan of global corporations [39]. This extension of the time horizon and the participation of the C-Suite, aligned with the integration in the supply chain, tend to confer to S&OP a strategic role in the company [45, 46].

**Fig 2:**
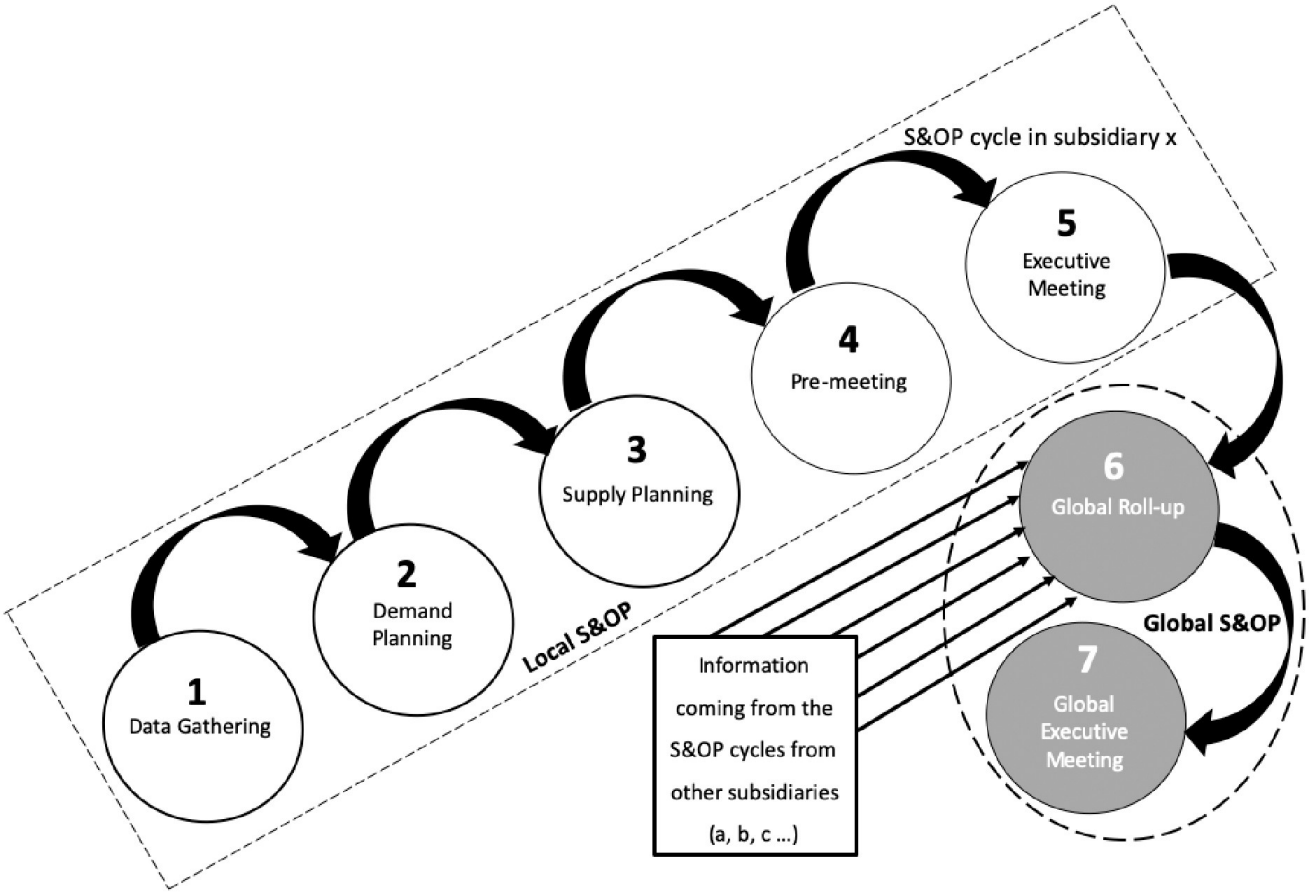
S&OP local and global process steps (Adapted from Wallace and Stahl [26]).

Fig 2 depicts these process steps, following Wallace and Stahl’s [26] framework that embraces the traditional five steps and introduces the two additional steps for consolidated global planning.

The first five steps have been analysed and explored in different empirical studies offered in the S&OP literature (e.g., [11, 40, 42]). Although steps six and seven have been presented in the literature (e.g., [26, 45]), they have not been the scope from scholars in investigations within real-life settings [20, 24], leaving a research gap to be investigated [19], which is addressed within this paper.

## Research method

**Fig 3:**
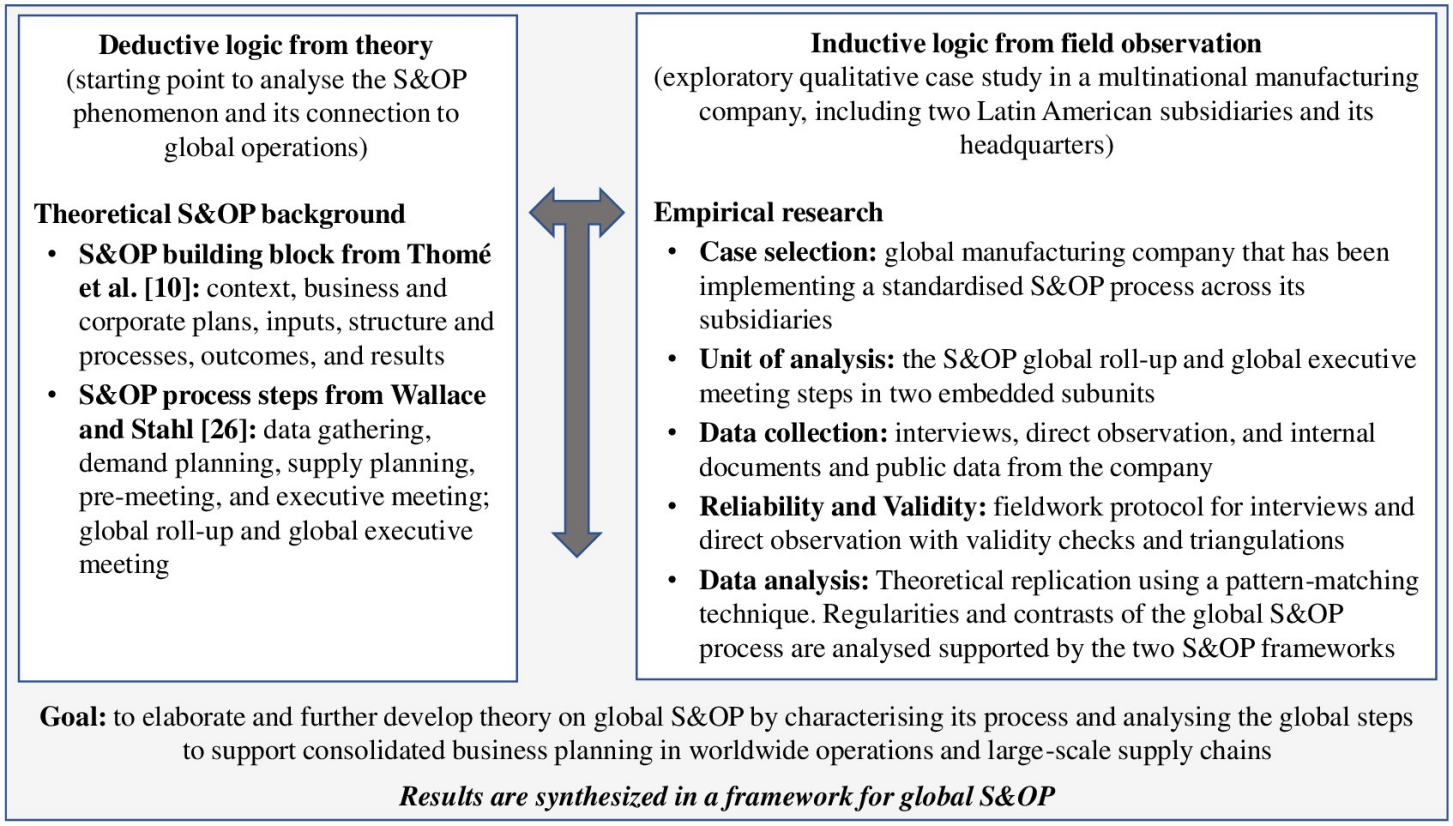
Research roadmap applying an abductive approach.

The extant literature on S&OP guided the development of the study’s research questions, the purpose and general objective, the case selection, and the data gathering method choice, following Yin [47]. The research initiates with a deductive logic [47], having the literature body on the theme as the starting point to analyse the S&OP phenomenon and its connection to large-scale supply chain and global operations. Two suitable S&OP frameworks were deduced from the theoretical S&OP background (i.e. Thomé et al. [10] and Wallace and Stahl [26]). As the knowledge in the literature about global S&OP is scarce, an exploratory qualitative case study is conducted, structured and guided by the offered frameworks to advance theory. The research applies an abductive approach. It combines the deductive logic from theory and inductive logic from field observation, in an attempt to elaborate further on theory on global S&OP, in a manner consistent with Ketokivi and Choi’s [33] strategy for theory elaboration in case study research. By building upon existing frameworks, the research applies the perspective of Meredith‘s [32] theory development approach aiming to advance the understanding on global S&OP, in the sense recently called for by Kreuter et al. [18]. Fig 3 synthesises the research approach in a comprehensive roadmap.

### Case selection

The study adopted a purposive sampling aiming at exploring an exemplar case of a single embedded case study design [47]. Exemplar companies adopting and documenting S&OP global process steps [26] are useful to explore new processes and benchmarking opportunities (e.g. [48]). The case company studied within this research is a global manufacturer that has been implementing a standardised S&OP process across all subsidiaries, including the two (embedded) sub-units in Brazil and Mexico. The company’s successful global S&OP program offers a unit of choice for an exemplar case providing an exclusive arena for the research.

### Unit of analysis and its motivation

The unit of analysis is the S&OP global roll-up and global executive meeting steps in the two embedded subunits (the Brazilian and Mexican subsidiaries). Four main criteria guided the choice of subunits. First, the S&OP processes in the two locations are representative of the global S&OP implementation company-wide, thus conferring conceptual representativeness to the sample [47]. Second, both subsidiaries have an established S&OP process with the traditional five-step cycle approach and an integration of their processes with the headquarters (sixth and seventh steps). Third, these subsidiaries are the most significant operations in Latin America, contributing together with more than 70% of the regional revenue. Fourth, Mexico and Brazil are the two biggest countries regarding territory, population, gross domestic product, and market size in the region, conferring additional relevance to the study of S&OP in global operations in emerging markets. The choice of a single case allows for more in-depth analysis [49] but it is also acknowledged as a limitation for testable theory elaboration, as it might result in limited external validity of single-case research [47, 50, 51]. The inconvenience of single-case research is somehow diminished by the fact it contains two “mini-cases” embedded (Brazilian and Mexican subsidiaries). The use of embedded units in single-case research is an attenuating factor for single-case research limitations in theory elaboration research, as indicated earlier by Dyer and Wilkins [51] in a rebuttal to Eisenhardt [50].

### Data collection

During the data collection, all institutional and individual data were anonymised to protect confidentiality, in accordance with recognised practices to protect human subjects and third parties for anonymity and confidentiality. For this reason, there will not be a disclosure of the case study database to the public domain. Additional relevant information could be disclosed on an as-needed basis upon request to the corresponding author. Verbal and written informed consents were given by personnel involved in the research. The appropriate STROBE guidelines for observational studies were followed to ensure transparency. The extensive field research to collect information consisted of interviews, direct observations, internal documents, and public data from the company. A semi-structured questionnaire was designed to support the interviews [47], adapted from Manuj and Sahin [52], which is presented in S1 Appendix. Interviews were conducted face-to-face following a protocol and questionnaire (approximately one hour to one hour and a half each). The questions were a guide to ensure that all topics of interest were covered. Although some questions had a yes/no format, interviewees were prompted to explain their answers further. Additional questions were made when necessary. Open-ended interviews were scheduled to complement and clarify the information obtained when needed. Whenever an interview revealed any contradictory data, the authors of this paper went back to the respondents to understand the fact aiming to achieve consensus. Data collected included meeting minutes, KPI dashboards, meeting calendars and attendance lists, action plans, meeting presentations, ERP system reports, and S&OP audit documents, among others. In addition to the interviews, the researchers had access to the subsidiaries and headquarters sites and personnel, including meeting attendance and day-to-day observation of the activities, allowing an in-depth study of the phenomena.

In total, 18 executives were interviewed: nine from Brazil, eight from Mexico and one from the headquarters in the USA. In Brazil, the interviews covered the functions of supply and S&OP manager, supply planner, demand planning and sales administration manager, sales director, supply chain director, manufacturing director, production planning manager, finance manager, and general manager. In Mexico, the interviews covered the functions of S&OP managers (two managers, each one responsible for different product lines), sales director, supply manager, purchasing planner, financial planning manager, supply chain director, and general manager. Interviews with the global S&OP leader happened during his visits to the subsidiaries and complemented by e-mail, and by phone calls.

Direct observation was also an important instrument for collecting data with one of the researchers taking part in the meetings of the S&OP process steps in both subsidiaries. The observational guideline adopted for data collection and analysis is presented in Table 1 in S2 Appendix. Field observations covered at least one year and a half in Brazil and two years in Mexico, comprising the period of global implementation when procedures were standardised, subsidiaries’ personnel were trained, and routine S&OP procedures were audited.

### Reliability and validity

Research reliability was strengthened with the use of a fieldwork protocol for interviews and direct observation, consistent with Yin’s [47] guidelines. Documents and internal reports were also verified and analysed as evidence of the practices performed in the subsidiaries. Validity checks and triangulations were accomplished by comparing answers from interviews with different professionals with field observation notes, internal documents, and comprehensive public data from the company.

### Data analysis

Theoretical replication using a pattern-matching technique [53, 54] was adopted for the analysis. Consistent with Almutari et al. [55], the pattern-matching technique embraced the phases of (i) stating the expected patterns based on the theoretical S&OP frameworks identified; (ii) testing the empirically found patterns for each S&OP design parameter in all steps of the process; (iii) providing advancements of the original theory and developing research outcomes as regularities and contrasts between theory and observation. The regularities and contrasts of the global S&OP process in the subsidiaries and corporate headquarters were systematically analysed, guided by the two identified S&OP frameworks. Wallace and Stahl’s [26] framework was applied to analyse how the process steps are conducted to move from a local to a global S&OP scale. Thomé et al.’s [10] framework aided in obtaining an S&OP understanding through analysing the main building blocks and design parameters. The combined use of these two frameworks enabled to answer the two research questions. The obtained research findings are synthesised into a novel framework and contribute to the elaboration and development of theory on global S&OP [32, 33].

## Local and global steps of the S&OP process

This section presents the local and global steps of the S&OP process in the embedded sub-units (the Brazilian and Mexican subsidiaries) and their connection with the multinational manufacturing company’s headquarters in the USA. It investigates how the S&OP process is performed for global operations, addressing RQ1.

The studied multinational manufacturing company is present in more than 50 countries. It produces and commercialises a wide variety of goods of recognised brands globally, employing around 20,000 people worldwide with revenues of approximately US$ 6 billion annually. Its corporate plan is deployed by the headquarters in the USA to all subsidiaries worldwide with central guidelines and objectives. Local plans and budgets are developed accordingly and agreed after a few negotiation rounds between each subsidiary and the global headquarters. All subsidiaries’ plans are consolidated at the corporate level, building the agreed-upon company’s business plan.

The multinational manufacturing company decided to adopt S&OP globally as a way to organise, integrate and monitor its efforts in the different subsidiaries around the world. The process was not new in the company as some subsidiaries already had a solid S&OP process and metrics implemented (e.g., USA, Canada, UK, France, Brazil, Mexico) but many others were still in early implementation stages. Therefore, the multinational manufacturing company decided to create a project and a global S&OP team to standardise and to deploy the process to all subsidiaries worldwide, to train the people, and to assess the implementations with periodic audits.

The Brazilian and the Mexican subsidiaries run an S&OP process, following similar steps to the ones described in Wallace and Stahl [26]. The process has a determined meeting calendar, agenda and minutes; participants with clear roles and responsibilities; and defined metrics. S&OP is a priority for the multinational manufacturing company, the local management teams sponsor the process in both countries, and thus S&OP activities are executed with good collaboration among the functional business areas. The supply chain director has the overall responsibility for the S&OP process, supported by the general manager and the other directors. These two subsidiaries have relaunched their S&OP, adjusting it to comply with the new standard and obtained good S&OP scores in the first audits, reflecting adherence to the process. The Brazilian subsidiary commercialises around 6500 stock-keeping-units (SKUs), delivering to approximately 8000 local customers, with 1400 employees, being responsible for close to 40% of the Latin American annual revenues. Around 65% of the portfolio is locally manufactured in two owned plants. The remaining is imported from several countries. The subsidiary has two distribution centres (DCs) attending local customers and exporting to 30 countries. The manufacturing strategies are make-to-stock (MTS) and buy-to-stock (BTS), based on the sales forecast. The Mexican subsidiary commercialises around 4000 SKUs, delivering to approximately 2000 local customers, with 150 employees, being responsible for one-third of Latin American annual revenues. The sourcing is complex as almost the entire portfolio is imported from different countries, stored in one DC, with no local manufacturing plants directly managed by the Mexican subsidiary, operating under a strategy of BTS based on the sales forecast. The organisation uses a postponement strategy to increase flexibility and reduce inventory, packing 25% of the portfolio at DC.

**Fig 4:**
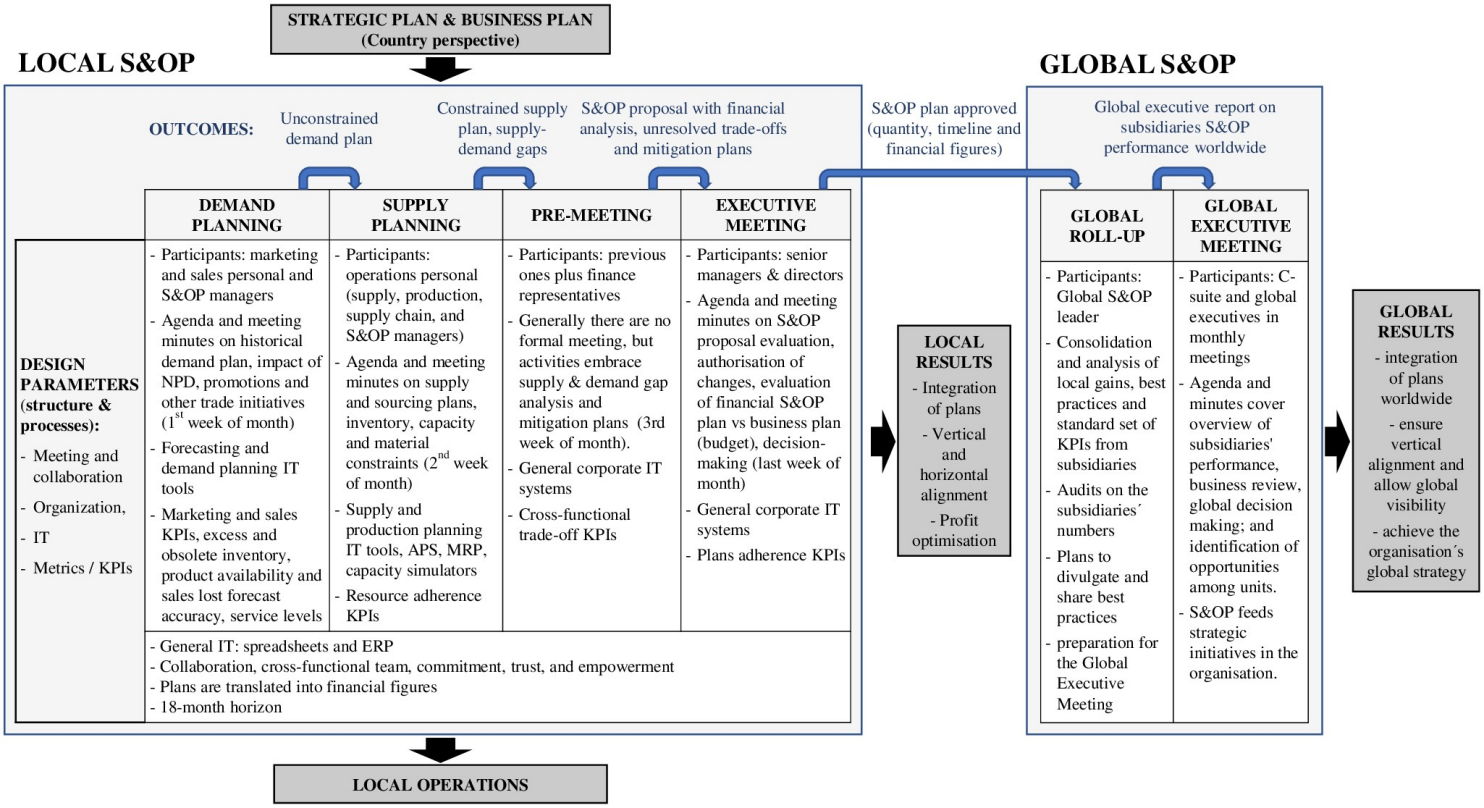
Global S&OP framework.

The S&OP local and global steps of the studied company are presented next, guided by the Global S&OP framework offered in Fig 4.

The framework combines both perspectives provided in Thomé et al.’s [10] and Wallace and Stahl’s [26] frameworks with the case study findings. It presents how the main S&OP building blocks, described by their design parameters, are characterised in each S&OP process step, embracing both local and global scopes. The framework embraces the outcomes, reflecting the output obtained in each process step, and results obtained in the local and global phases. Data gathering is not considered a step of the process because the recollection of information and the ERP system feeding is an automatically conducted activity at the case company, starting at the beginning of the month and continuing daily as new business transactions are registered. The local and global S&OP process steps are presented in the following subsections.

### Demand planning

In both subsidiaries, sales and marketing personnel generate the demand plan at aggregate and SKU levels with an 18-month horizon. The plan is translated into financial figures. Sales and marketing aim to deliver the committed budget. Marketing provides new product development (NPD) and promotion information and reviews the portfolio of products. Demand planning occupies part of the marketing and sales meeting held in the first week of the month, during which the S&OP managers in each subsidiary make a presentation covering historical data and KPIs (e.g., excess and obsolete inventory, product availability and sales lost, forecast accuracy and service levels), and review the meeting minutes. In Brazil, there are four sales directors responsible for different product lines. The demand planning & sales administration manager and his team generate the forecast by SKU, using dedicated software. Inputs gathered from sales and marketing (e.g., new product launches and promotions) are also included to produce the unconstrained demand plan. The forecast accuracy has been around 80% consistently. Factors favouring results are a broad portfolio, a large client basis, the usage of a demand planning tool by skilled professionals and the coordination performed by the demand planning & sales administration manager. The supply manager in Brazil is also the S&OP manager. He attends the demand meeting, receives the unconstrained demand plan from the demand planning & sales administration manager and takes it to the supply team.

In Mexico, five sales directors are responding to different product lines and distribution channels in a hybrid system. Two S&OP managers prepare the forecast by SKU, using spreadsheets, based on historical data from the ERP. During the demand meeting, they confirm the plan by SKU and by value with sales and marketing teams to produce the unconstrained demand plan. Forecast accuracy has improved but still struggles around 60%. The Mexican subsidiary has had challenges to deliver the budget and has impacted forecast accuracy and inventory planning due to unplanned sales initiatives. During this research, the S&OP managers were moved from the operations to sales, replicating the Brazilian structure. They became demand planning & sales administration managers, responsible for building the demand plan and its follow-up. The positive impact of this change was the increase in the demand plan ownership from sales. The outcome of the S&OP process, in both subsidiaries, was an unconstrained demand plan.

### Supply planning

In both subsidiaries, the unconstrained demand plan is the main input to supply planning. The supply meeting is held in the second week of the month with the presence of the supply chain director. During this meeting, respectively the Brazilian supply and S&OP manager and the Mexicans demand planning and S&OP managers make a presentation of historical data and KPIs (e.g., resource adherence). The supply capacities are previously simulated. In the meeting, the feasible supply plan and alternatives to mitigate gaps are costed out and discussed. The supply plan is translated into financial figures. The supply team seeks to deliver the budget, while finance may be asked to assess and approve mitigation plans involving extra costs going beyond the decision-making autonomy of the functional managers. In Brazil, the supply and S&OP manager with his team is responsible for coordinating the supply plan construction. They provide to two local plants a production master plan to be simulated and a sourcing plan that takes into account inventories on-hands, in-transit, and the inventory policy. The production planners use a Material Requirement Planning (MRP) system to generate purchasing and production orders. In the biggest plant (a thousand employees, hundreds of machines, thousands of SKUs), an advanced planning system is applied for simulations, in production scheduling, and shop floor control. The smaller plant (half a dozen machines, around 20 employees, less than a hundred SKUs) uses MRP and spreadsheets to plan and control. The production planners work together with local purchasing sector and international commerce sector to confirm raw material and packaging material availability. The supply and S&OP manager also works together with the international commerce sector to confirm with global suppliers the new orders acceptance, the current orders status and the expected arrival dates of finished goods. The Mexican supply plan is a sourcing plan. The supply manager and his team work together with the international commerce sector to confirm with suppliers the acceptance of new orders and status and expected arrival dates of the current ones. The supply team generates a sourcing plan based on inventory on-hands and in-transit, considering inventory policies by SKU.

The outcome of this step is a constrained supply plan (by SKUs and value). Gaps to deliver the demand plan are identified with respective mitigation plan proposals when approval is beyond the autonomy of the functional managers’ decision-making.

### Pre-meeting

In both subsidiaries, this step usually does not include a formal meeting. It involves the participants from the previous steps and finance representatives in decisions associated with supply and demand gaps and mitigation plans during the third week of the month. In Brazil, the supply and S&OP manager talks to the demand planning and sales administration manager to communicate the supply plan with the best efforts to mitigate all gaps as well as the potential downsides by SKU. Exhaustive conversations are held before this point with manufacturing plants and suppliers to deliver the demand plan. Alternative measures that go beyond the supply team’s and supply chain director’s autonomy are taken to the executive meeting. In Mexico, the supply manager has a similar conversation with demand planning and S&OP managers. Metrics in this step embrace cross-functional trade-off measures (demand vs production and strategic and tactical alignment). As in the other steps, there are transparency, commitment, trust, and collaborative spirit among all the functional business areas involved, which empowers S&OP in the company. The outcome of this step is an S&OP proposal (by SKUs and value) with financial analysis, unresolved trade-offs, and mitigation plans.

### Executive meeting

The executive meeting is performed in both subsidiaries with the presence of the general manager and directors. The focus is on the S&OP proposal evaluation, authorisation of changes, evaluation of financial S&OP plan vs business plan (budget), and decision-making. There is a business review in the last week of the month, and the executive meeting takes part of its time. During this step, the supply and S&OP manager in Brazil and the demand planning and S&OP managers in Mexico present the S&OP plan proposal in units per SKU and value, with an 18-month horizon. The S&OP plan is compared to the budget with a risk analysis, seeking to deliver the revenues committed with the headquarters. When plan and budget do not match, the focus moves directly to analyse the recommended alternatives to close the gaps for approval. Even though the objective is analysing the following months, the current month is also discussed. As part of the presentation, KPIs are reviewed, focusing on plans adherence (e.g., supply vs demand; S&OP finance vs financial plan and budget). Four sales directors, a supply chain director, two manufacturing directors, three marketing directors, one human resources director, one finance director and the general manager, compose the Brazilian management team. In Mexico, the management team is composed of five sales directors, one trade-marketing director, two marketing directors, one human resources director, one supply chain director, one finance director, and the general manager. In addition to the management team, the S&OP managers in each subsidiary and special invitees, attend the executive meeting. The outcome is the approved S&OP plan for the subsidiary, which is shared with the headquarters in the USA.

The two subsidiaries have launched hundreds of SKUs every year and increased the customer basis to grow their businesses. The Mexican subsidiary overcomes two merger processes in a short period. During the mergers, turnover was high, two local manufacturing plants were closed, and the entire portfolio became imported. The extensive observations indicate that S&OP has been key to manage the complexity and dynamic changes in both subsidiaries. Local results have revealed direct gains as profit increase as well as a successful integration of plans among the different functional areas and vertical and horizontal alignment.

### Global roll-up

This step is coordinated by the global S&OP leader, a director from headquarters responsible for deploying and standardising the process worldwide. Within this step, information from the subsidiaries is gathered, consolidated, and analysed, which is then included in an executive report for the global executive meeting. The report indicates local gains in the subsidiaries (e.g., improved inventory management, forecast accuracy, cost reduction, service level increase, among others) as well as their implementation progress, best practices, and KPIs. There is a standard set of KPIs that all subsidiaries worldwide have to keep track and report: perfect orders, line fill, forecast accuracy, distribution and transportation costs, inventory value (gross, raw material, work in progress, excess and obsolete), inventory turns, inventory days-on-hands, total SKU count, SKU ABC curve and the company’s S&OP assessment score. The S&OP assessment score measures the adherence to the multinational manufacturing company’s standard S&OP process each subsidiary’s process achieved and is determined in periodic audits. These metrics are presented in a scorecard and are shared with the corporate headquarters periodically, during global roll-up. KPIs and best practices are also shared among the subsidiaries, providing an additional gain for local operations, as also pointed out in Basu [28] and Lapide [29], and are used to orient trainings and workshops with the subsidiaries.

### Global executive meeting

In this final process step, the decision-making moves up to the C-Suite and global executives. Therefore, the whole process becomes more focused on strategic issues, as foreseen in VICS [45] and in Baumann [46], such as the balance of capacities and portfolio of products beyond countries and business units’ frontiers. This meeting aims to monitor and analyse the subsidiaries S&OP performance worldwide on a monthly base as well as to support and evaluate the S&OP global deployment. Best practices, local gains, and KPIs are discussed, and business opportunities are identified, resulting in decision-making and requests for additional information or corrective action plans if problems, conflicts and non-expected results are identified. The possibility to understand the big global picture and make decisions accordingly is considered by the C-Suite as a source to obtain competitive advantage. It aims to build a broad customer portfolio attending global demand and supply, which allows the company to switch volumes when product sales drop in one region or to find production or sourcing capacity to explore new markets, to maintain profitability. This is in line with Thomas et al. [56] regarding successful manufacturing organisations.

The global results achieved with the S&OP process in the multinational company were synthesised by its global S&OP executive leader as: (i) harmonise the integration of plans worldwide, (ii) ensure vertical alignment and allow global visibility and (iii) achieve the organisation´s global strategy.

## Consolidation and integration of local S&OP into a global plan

**Fig 5:**
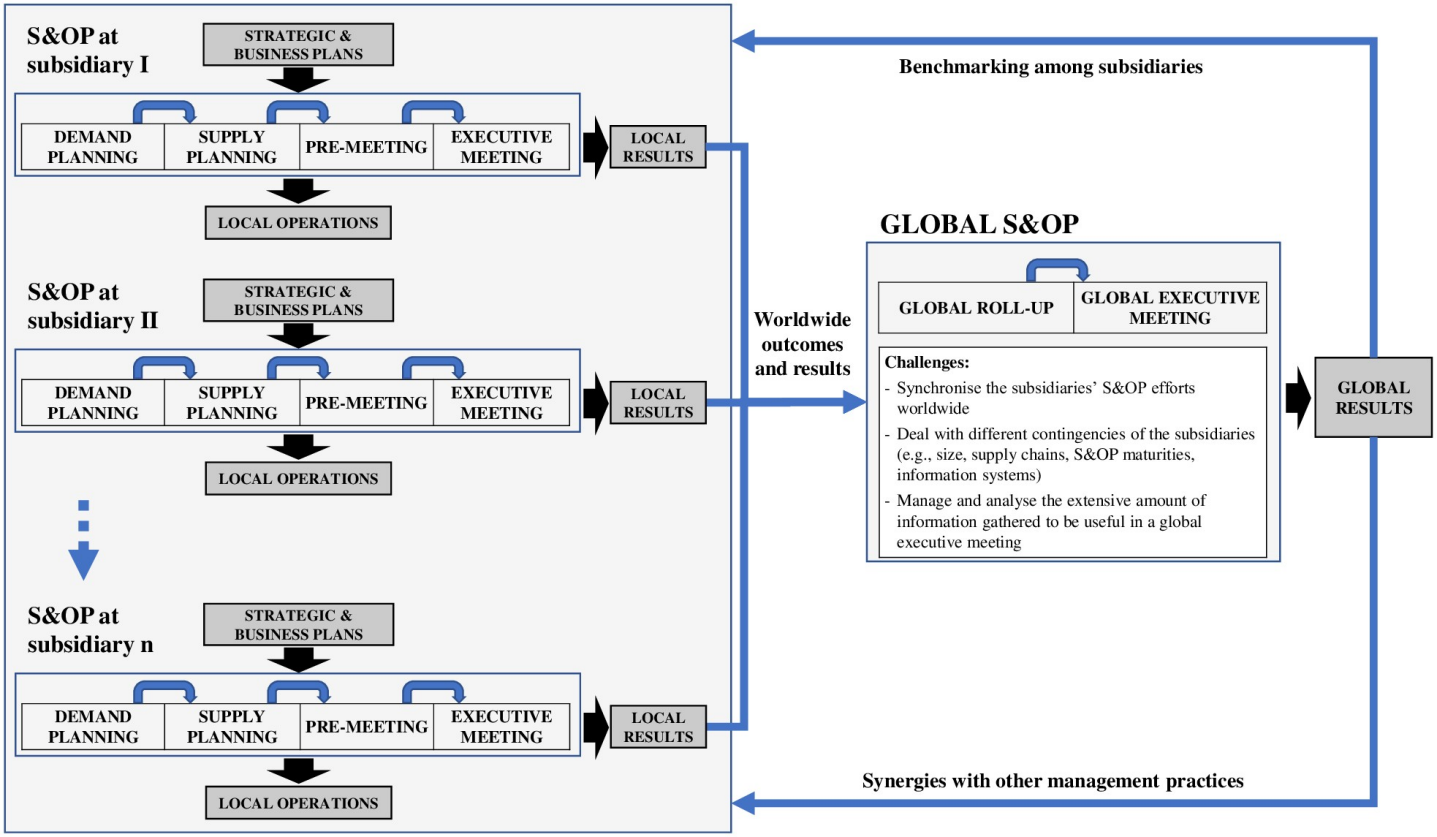
Global S&OP at the case study company.

This section investigates challenges and global results associated with the consolidation and integration of the different local S&OP worldwide, addressing RQ2. Fig 5 synthesises findings, which are further discussed in the following two subsections.

### Challenges for the global S&OP

The global S&OP implementation results are considered satisfactory within the multinational manufacturing company. However, the company is facing different challenges that need to be surpassed to reach the full benefits of global gains. The company faces difficulties of gathering S&OP information in a comparable format from around 50 countries with many subsidiaries using different systems (software and versions). The data gathering and its consolidation have been a severe challenge and an issue not completely resolved yet. Different ERP systems and versions are running in the countries, and part of the information has to be edited and consolidated manually in spreadsheets before each subsidiary sends it to the headquarters. This jeopardises the accuracy of the information, especially when it comes from small subsidiaries. The company’s S&OP manual supports the data harmonisation, as it shows which KPIs to use, how they are calculated, and examples of presentation formats. However, due to a lack of personnel and training, it is not always completely followed. The company has recognised the inadequacy of their information systems to be a central problem of their global S&OP. Therefore, it currently aims to integrate its worldwide information systems within a process of bringing together all sub-systems to deliver the intended functionality, similar as described in Ghobakhloo [57]. To get the benefits from their implementation, the existing management systems and standard processes ought to be integrated into a single system, in a sense discussed in Zutshi and Sohal [58]. The company has decided to implement one ERP as a worldwide standard, which is seen as an enabler for the processes to get standardised and the information to become more reliable and timely available.

Moreover, a challenge lies in the interpretation and use of the information gathered within the global S&OP process. The amount of detailed information available to review is vast, and the headquarters aims to avoid a “data overload” [31], as the C-suite does not want to micromanage the subsidiaries. The company rather sees its global S&OP information as an opportunity to get a worldwide picture that facilitates the development of strategic initiatives to improve the overall business. Herein, a particular challenge lies in the comparison of results from the various subsidiaries due to their different S&OP maturity stages (e.g., while some subsidiaries are running their S&OP effectively, others are still struggling to implement the first stages) and differences in terms of their infrastructure, size, and complexity. To illustrate the problem, Brazil and Mexico, each one considered alone, are bigger businesses than all the other countries in Latin America together. At the same time, the USA’s sales are more than ten times the entire Latin America regional sales. Brazil has a country size comparable to the USA and a similar number of customers but, instead of shipping full truckloads as in the USA, the Brazilian order profile is similar to United Kingdom’s, primarily small and medium orders, less than a truckload. Some subsidiaries have manufacturing plants and carry inventory to export for a transference cost while small subsidiaries are just commercial units selling profit margins and carrying a low inventory. Furthermore, smaller subsidiaries, scarce in resources, have difficulties to generate the information for their S&OP KPI dashboard. The information from their local S&OP process is not entirely reliable with frequent miscalculations leading to misinterpretations. Additional challenges that make it difficult to compare the subsidiaries’ performances are related to reporting standards within global, large-scale supply chains. For instance, subsidiaries with local plants also carry extra inventory (raw materials, work in progress, and finished goods) to supply other regions. They are responsible for production and inventory costs, but export to the destiny subsidiary for a transfer price. The profit appears in the result of the subsidiary that imports, which has a leaner structure when it sells in its local market. The challenges to consolidate the subsidiaries’ results globally demonstrate the importance of considering this financial perspective within the global S&OP.

The studied company views the implementation of the global S&OP process as a permanent learning effort. It is continuously elaborating how the global executive meeting should deal best with the challenges noticed above. Nevertheless, it is considered essential that the C-suite demonstrates the importance of the global S&OP process for the company by dedicating time, attention, and sponsorship to it. This is seen as more important than understanding in depth the details, which are mainly handled by executives in the subsidiaries locally.

### Global results

Steps six and seven of the global S&OP have been important to monitor the S&OP implementation progress in each subsidiary on a regular basis. The company developed a standard S&OP KPI dashboard, which is used by the subsidiaries worldwide. Every subsidiary has to report its results monthly, using performance indicators that are calculated in the same way. The KPIs cover different aspects such as costs, service levels, inventory data, forecast accuracy, and the S&OP assessment grade. Best practices and local gains are also reported within the global roll-up step. These global reports are shared and have generated valuable insights that resulted in benchmarking possibilities among subsidiaries. Benchmarking was not expected and planned in the original global S&OP project, but was considered an opportunity to be explored and a beneficial outcome of the global process. For instance, best practices of superior performance subsidiaries could be identified by assessing their forecast accuracy, service level, and S&OP assessment score. Moreover, advanced comparisons among portfolios of products in the different regions have become possible. Although corporate marketing had been working with the local subsidiaries’ teams already to explore opportunities related to portfolio management among regions, the global S&OP process now makes this information available to the C-suite periodically. The same applies to the current spare capacity analysis by corporate operations at local levels, which is now discussed more often at headquarters’ global meetings. Facilitated by the global S&OP, the corporate headquarters and subsidiaries are learning how to handle external and internal benchmarking, as envisioned in Basu [28], setting up performance improvement initiatives, and obtaining synergies with other management initiative and practices, as anticipated in VICS [45]. Although not being part of the global executive meeting discussion, the additional synergies facilitate the alignment of local and global expectations, reducing the “fight over decision rights” presented in Alexander [31] among the global headquarters and the local subsidiaries. For instance, the global sourcing team in the headquarters is now using data from the S&OP of the subsidiaries to analyse global versus local procurement.

Moreover, global S&OP related project to implement one ERP as the company’s global standard created several synergy effects within the company. By sharing the same ERP system worldwide, the company advanced towards the complete integration of its units, standardising processes around the world, and automating reports and KPIs’ calculation. Another synergy is associated with the analysis of the company’s different business units, which are composed by the related family of products with similar characteristics and brands. The business units have representatives in the subsidiaries regionally and globally at the headquarters, looking specifically at product lines and brands. The business units can now utilise information from the global S&OP to help build their strategies and develop targets, projects, and action plans.

The C-suite is seeing several gains from the global roll-up, as envisaged in Lapide [29] and Seeling et al. [24] for global S&OP regarding the “harmonisation” of plans among different subsidiaries and the increased visibility of the global operations. Locations with excess and obsolete products and plants with high spare capacities, affecting the return on assets, are brought to the attention of the C-suite, provoking reactions and speeding the development of solutions. Requests for assessments about plant capacity constraints and opportunities to increase portfolios in new countries and questions about service level issues are addressed with priority by the subsidiaries as the C-suite now sees them and can facilitate their implementation. The global S&OP provides the C-suite with a more holistic view, enabling to analyse the total company results and initiating global actions for change. This, for instance, allows to shift production capacities among the subsidiaries according to the organisation’s global strategy, as envisioned in Lapide [29] and Nemati et al. [25]. Furthermore, the global view implies strategic implications for S&OP that are not observed in local approaches limited to the first five cycle steps. Decisions affecting a strategic planning horizon have gained more attention in the Brazilian and Mexican subsidiaries’ S&OP, as now the local executives know that they are being reported to the company’s headquarters. Regional vice-presidents, directors, and functional managers are now questioning S&OP results more frequently, and extraordinary results are noticed and discussed.

## Theoretical advancements and practical implications

The empirical evidence gathered within this research served to analyse and evaluate the global S&OP process in the multinational manufacturing company, applying two established S&OP frameworks as guidance (i.e., Thomé et al. [10] and Wallace and Stahl [26]). The novel framework, generated on that basis, goes beyond the traditional local S&OP approach and embraces a global perspective. Thereby, this paper contributes to the cycle of describing, explaining, validating, and expanding existing knowledge, enabling the theoretical advancement of the research domain [32], and conduces to theory elaboration using the logic of abduction, as defined by Ketokivi and Choi [33]. On the one hand, the research findings extend the accumulated empirical evidence on S&OP, enabling the progression and maturation of this emerging topic [32, 33, 59, 60]. On the other hand, by applying proven frameworks within a case study setting, this research enhances the external validity and generalisation of S&OP findings, and contributes to theory development by building a continuous line of research within the domain [32]. The paper’s findings contribute to developing a domain-specific theory on S&OP, in a sense of the middle-range theorising strategy from Sociology [61], addressing a recent call to extend the understanding of the S&OP phenomenon in this vein [18, 19].

Moreover, the research findings demonstrate the cross-disciplinary nature of S&OP, highlighting the vital role and interdependency of human behaviour and IT for implementing global S&OP processes. To capture and understand those aspects, investigations should not be limited to a pure focus on the process itself. This implies the need to go beyond the traditional Operations Management perspective taken in the current literature by applying concepts and theories borrowed from other disciplines to advance the S&OP understanding. As demonstrated by Goh and Eldridge [19], most empirical research in S&OP is not based on established concepts and theories with ample space for more theory-informed S&OP investigations. Chicksand et al. [62] showcase the benefits of applying a diversity of theoretical lenses to the a-theoretical emergent management topic of purchasing and supply chain management. Similarly, by emphasising the multidisciplinary nature of the nascent topic of S&OP, the research findings of this paper underline the substantive gains to be sought by applying different theoretical lenses to the understanding of global S&OP practices. The results from this exemplar case study particularly indicate the use of theories from the germane fields of Economics, Psychology, and Information System as a case in point for understanding S&OP management practice. For instance, from the discipline of Economics, the theory of transaction cost economics posits that firms may incur costs whenever a transaction is managed, due to the actors`bounded rationality, opportunism, and comparative costs between hierarchies and the market [63]. This is highly relevant for global S&OP processes embracing worldwide operations and large-scale supply chains, like the ones investigated in this paper, where a focus on reducing costs of the different S&OP transactions or mitigating eventual opportunism in the balance of supply and demand could generate significant benefits, depending on context. Additional applications of concepts from Economics should be pursued further in the future. Furthermore, perspectives from the Psychology and Information Systems disciplines could serve to understand human behaviour and the role of IT within S&OP. The case study findings revealed that interpersonal trust, openness, collaboration, and an organisational culture, in which the business functions and the employees accept their responsibilities, is required for a successful implementation of global S&OP (e.g., functional teams must share information; there must be a save environment that allows to discuss and disagree in a professional manner; broad cross-functional communication between different cultures is necessary; joint work to get consensus and generate plans to achieve the company’s objectives requires a common mindset). First attempts to understand the psychological factors behind such aspects have been conducted by Ambrose and Rutherford [11] regarding the application of group effectiveness theory [64] Hackman et al. 2000) and Ambrose et al. (2018) concerning the application of social identity theory [65, 66] both stemming from Psychology. In line with the enabling role of IT identified in this paper, Kreuter et al. [22] have presented an approach for the development and implementation of S&OP designs, ingrained in enterprise architecture management [67], an approach borrowed from the Information Systems discipline. Future research should continue investigating S&OP from a multidisciplinary perspective to advance the domain’s theoretical understanding.

Besides the theoretical contributions, this paper offers practical implications that can assist executives, especially from multinational manufacturing companies, in their efforts to consolidate global planning. The global S&OP framework characterises the process for worldwide operations and large-scale supply chains, addressing a recent gap that inhibits successful practical implementations. The framework embraces the interplay between the local and the under-researched global S&OP steps to support consolidated business planning, and offers their main design parameters, incomes, and outcomes. Especially, the lessons learned from designing and implementing steps six (global roll-up) and seven (global executive meeting) are paramount for practitioners operating global planning in multinational companies. The case study demonstrates the importance of the C-suite’s sponsorship to the global S&OP implementation to keep all subsidiaries focused on the process, which reinforces findings from Wallace and Stahl [26]. The sponsorship also speeds up the S&OP adoption and enhances its results. The results obtained by the studied company (e.g., inventory reduction, forecast accuracy increase, service level improvement) and the company benchmarks indicate that integrating the local five-step S&OP process with the two global steps has been key to successfully manage the organisational complexity associated with the different geographic regions offering a broad product portfolio. Another practical implication, not investigated previously in the S&OP literature, regards to the worldwide integration of information systems to overcome the difficulty of gathering and managing the myriad of data, coming from subsidiaries along the globe that use different reporting systems. The experience gained from the path of introducing a unified dashboard, in which all subsidiaries report their results monthly, and the subsequent implementation of a standardised global information system is of high relevance for improving the global S&OP roll-up in multinational companies. Moreover, practitioners can learn that the global S&OP process enables additional benefits and synergetic effects that go beyond the ones specifically quantified as a result of the global executive meeting, including the timing of product launches across countries, learning from experiences in different regional markets, and exploring business opportunities across the globe. Finally, the implementation of the global S&OP steps has encouraged the sharing of best-practices worldwide and positively impacted the company culture, as emphasised by the executives from the different subsidiaries and headquarters.

## Conclusion

The S&OP research body has grown significantly with advances in the understanding of the process within a local perspective, generally concentrated within a business unit or limited to a country frontier. As companies extend their operations and plan integration efforts geographically, global S&OP emerges as a relevant research topic. This paper addresses this issue, contributing to an advanced understanding of how global steps are performed and what challenges and opportunities are associated with a worldwide S&OP implementation. The paper offers a unique analysis of the global S&OP of a multinational manufacturing company, embracing two of its main Latin American subsidiaries and its corporate headquarters in the USA. The analysis is structured and guided by a framework for global S&OP, developed based on the theoretical background and the case study findings. This novel framework contributes to theory on global S&OP through case study research, in a manner consistent with Meredith’s [32] and Ketokivi and Choi’s [33] for empirically-grounded theoretical elaboration and development.

This paper contributes to the literature in S&OP in different ways. First, it examines the under-investigated global steps, going beyond the standard regional S&OP five-step cycle. Findings reveal details and novel outcomes of these global steps, going beyond the general S&OP plans approved by the subsidiaries. The outcomes embrace a standard set of KPIs adopted worldwide, evaluations of best practices, local gains among subsidiaries, and the identification of business opportunities. Moreover, the results from the global steps can feed strategic initiatives within the organisation. Second, the paper identifies challenges to implement and conduct a global S&OP. These include the need to synchronise the subsidiaries’ S&OP efforts, to manage the amount of information gathered worldwide, to deal with different contingencies of the subsidiaries, and to harmonise data from different information systems. Third, the paper sheds lights on the opportunities of a global perspective for S&OP. It not only confirms the envisioned opportunities offered in the literature, but it suggests additional ones as benchmarking among subsidiaries and synergies with other management practices. Fourth, evidence from the case study research indicates the gradual evolution from tactic to strategic planning when global roll-up and global executive meetings are stimulated. The focus of the global S&OP bends towards strategic rather than the tactical decision-making described in the traditional S&OP literature. The related additional benefits involve strategic organisational development aspects with overall business implications. Finally, the research findings present practical implications and contribute to the theoretical advancement of S&OP research, opening avenues for middle-range theorising, highlighting the cross-disciplinary nature of the domain, and discussing the use of concepts from different disciplines as Economics, Psychology, and Information Systems. Future research should also address the limitations of this study towards generalising its findings. The case study is limited to two subsidiaries in Latin America and the corporate headquarters in the USA of a multinational manufacturing company that produces and commercialises consumer goods. The generalisability of findings might, therefore, be limited to the analysed context (e.g., type of industry, the corporation’s S&OP maturity stage, and considered countries). Therefore, the functioning of S&OP in large multinational organisations and the deployment of a global roll-up and a global executive meeting steps should be investigated further in additional empirical studies evaluating different contexts.

## Supporting information

S1 Appendix (DOCX)

S2 Appendix (DOCX)

## References

[1] Fachini RF, Esposto KF, and Camargo VCB. A framework for development of advanced planning and scheduling (APS) systems in glass container industry. J. Manuf. Technol. Manag. 2018; 29(3): 570–587.

[2] Jonsson P, Kaipia R, Barratt M. The future of S&OP: dynamic complexity, ecosystems and resilience. International Journal of Physical Distribution and Logistics Management. 2021; 51(6): 553–565. 10.1108/IJPDLM-07-2021-452.

[3] Barbosa MW, Vicente ADLC, Ladeira MB, Oliveira MPVD. Managing supply chain resources with Big Data Analytics: a systematic review. Int. J. Logist. Res. Appl. 2018; 21(3):177–200.

[4] Ferreira FAL, Scavarda LF, Ceryno PS, Leiras A. Supply chain risk analysis: a shipbuilding industry case. Int. J. Logist. Res. Appl. 2018; 21(5): 542–55.

[5] Jonsson P, Holmström J. The future of supply chain planning: Closing the gaps between practice and promise. Int. J. Phys. Distrib. Logist. Manag. 2016; 46(1): 62–8.

[6] García-Villarreal E, Bhamra R, Schoenheit M. Critical success factors of medical technology supply chains. Prod. Plan. Control. 2019; 30(9): 716–735.

[7] Tuomikangas N, Kaipia R. A coordination framework for sales and operations planning (S&OP): synthesis from the literature. Int. J. Prod. Econ. 2014; 154: 243–262.

[8] Goh SH, Eldridge S. Sales and Operations Planning: The effect of coordination mechanisms on supply chain performance. Int. J. Prod. Econ. 2019; 214: 80–94.

[9] Lapide L. Sales and operations planning Part III: a diagnostic model. J. Bus. Forecast. 2005; 24(1): 13–15.

[10] Thomé AMT, Scavarda LF, Fernandez NS, Scavarda AJ. Sales and Operations Planning: a research synthesis. Int. J. Prod. Econ. 2012a; 138(1):1–13.

[11] Ambrose SC, Rutherford BN. Sales and operations planning (S&OP): a group effectiveness approach. J. Acad. Mark. Sci. 2016; 20(2): 36–60.

[12] Thomé AMT, Scavarda LF, Fernandez NS, Scavarda AJ. Sales and Operations Planning and the firm performance,” Int. J. Product. Perform. Manag. 2012b; 61(4): 359–381.

[13] Thomé AMT, Sousa RS, Carmo LF. The impact of sales and operations planning practices on manufacturing operational performance. Int. J. Prod. Res. 2014; 52(7): 2108–2121.

[14] Ben Ali M, D’Amours S, Gaudreault J, Carle MA. Integrating revenue management and sales and operations planning in a Make-To-Stock environment: softwood lumber case study. INFOR. 2019; 57(2): 314–341.

[15] Noroozi S, Wikner J. Sales and operations planning in the process industry: A literature review. Int. J. Prod. Econ. 2017; 188: 139–155.

[16] Kristensen J, Jonsson P. Context-based sales and operations planning (S&OP) research: A literature review and future agenda. Int. J. Phys. Distrib. Logist. Manag. 2018; 48(1): 19–46.

[17] Pereira DF, Oliveira JF, Carravilla MA. Tactical sales and operations planning: A holistic framework and a literature review of decision-making models. Int. J. Prod. Econ. 2020; 228: 107695.

[18] Kreuter T, Scavarda LF, Thomé AMT, Hellingrath B, Seeling MX. Empirical and theoretical perspectives in sales and operations planning. Review of Managerial Science. 2021a; (in press). doi: 10.1007/s11846-021-00455-y PMC8454961 34547059

[19] Goh SH, Eldridge S. New product introduction and supplier integration in sales and operations planning. Int. J. Phys. Distrib. Logist. Manag. 2015; 45(9/10): 861–886.

[20] Pedroso CB, Silva AL, Tate WL. Sales and Operations Planning (S&OP): insights from a multi-case study of Brazilian organisations. Int. J. Prod. Econ. 2016; 182: 213–229.

[21] Scavarda LF, Hellingrath B, Kreuter T, Thomé AMT, Seeling MX, Fischer JH, et al. A case method for Sales and Operations Planning: a learning experience from Germany. Prod. 2017; 27(SPE): e20162199. 10.1590/0103-6513.219916

[22] Kreuter T, Kalla C, Scavarda LF, Thomé AMT, Hellingrath B. Developing and implementing contextualised S&OP designs—an enterprise architecture management approach. International Journal of Physical Distribution and Logistics Management. 2021b; 51(6): 634–655.

[23] Lim LL, Alpan G, Penz B. A simulation-optimisation approach for sales and operations planning in build-to-order industries with distant sourcing: Focus on the automotive industry. CAIE. 2017; 12: 469–482.

[24] Seeling MX, Scavarda LF, Thomé AMT. A sales and operations planning application in the Brazilian subsidiary of a multinational chemical company. Braz. J. Oper. Prod. Manag. 2019. 16(3): 424–435.

[25] Nemati Y, Madhoushi M, Safaei Ghadikolaei A. Towards supply chain planning integration: uncertainty analysis using fuzzy mathematical programming approach in a plastic forming company. IJMS. 2017; 10(2): 335–364.

[26] Wallace TF, Stahl RA. Sales and Operations Planning The Executive’s Guide. TF Wallace & Co. 2006.

[27] Stentoft J, Freytag PV, Mikkelsen OS. The S&OP process and the influence of personality and key behavioral indicators: insights from a longitudinal case study. International Journal of Physical Distribution & Logistics Management. 2020; doi: 10.1108/IJPDLM-02-2020-0056

[28] Basu R. New criteria of performance management: a transition from enterprise to collaborative supply chain. MBE. 2001; 5(4): 7–12.

[29] Lapide L. Global S&OP: parsing the process. J. Bus. Forecast. 2011; 30(4): 15–18.

[30] Kelleher M. How to improve the global S&OP process: Hollister’s journey. J. Bus. Forecast. 2012; 31(1): 4–17.

[31] Alexander D. S&OP in the 21st Century. J. Bus. Forecast. 2016; 35(1): 10–34.

[32] Meredith J. Theory building through conceptual methods. *International Journal of Operations & Production Management*. 1993.

[33] Ketokivi M, Choi T. Renaissance of case research as a scientific method. JOM. 2014; 32(5): 232–240.

[34] Qi J, Ellinger AE. A conceptual framework of organisational orientation antecedents of sales and operations planning. in Stieler M (Ed.) Creating Marketing Magic and Innovative Future Marketing Trends. Cham, Switzerland, Springer. 2017; 1319–1329.

[35] Ivert LK, Dukovska-Popovska I, Kaipia R, Fredriksson A, Dreyer HC, Johansson MI, et al. Sales and operations planning: responding to the needs of industrial food producers. Prod. Plan. Control. 2015a; 26(4): 280–295.

[36] Ivert LK, Dukovska-Popovska I, Fredriksson A, Dreyer HC, Kaipia R. Contingency between S&OP design and planning environment. Int. J. Phys. Distrib. Logist. Manag. 2015b; 45(8): 747–773.

[37] Kathuria R, Joshi MP, Porth SJ. Organizational Alignment and performance: past, present and future. MD. 2007 45(3): 503–517.

[38] Thomé AMT, Sousa RS, Do Carmo LFRRS. Complexity as contingency in sales and operations planning. IMDS. 2014b; 114(5): 678–695.

[39] Kaipia R, Holmström J, Småros J, Rajala R. Information sharing for sales and operations planning: Contextualised solutions and mechanisms. JOM. 2017; 52: 15–29.

[40] Wagner SM, Ullrich KK, Transchel S. The game plan for aligning the organization. Bus. Horiz. 2014; 57(2): 189–201.

[41] Hulthén H, Näslund D, Norrman A. Framework for measuring performance of the sales and operations planning process. Int. J. Phys. Distrib. Logist. Manag. 2016; 46(9): 809–835.

[42] Grimson JA, Pyke DF. Sales and operations planning: an exploratory study and framework. Int. J. Logist. Manag. 2007; 18(3): 322–346.

[43] Hulthén H, Näslund D, Norrman A. Challenges of Measuring Performance of the Sales and Operations Planning Process. OSCM. 2017; 10(1): 4–16.

[44] Thomé AMT., Hollmann RL, Scavarda LF Research synthesis in collaborative planning forecast and replenishment. IMDS, 2014; 114: 949–965.

[45] VICS. Linking CPFR and S&OP: A Roadmap to Integrated Business Planning. Solutions. 2010; 1–24.

[46] Baumann F. The shelf-connected supply chain: strategically linking CPFR with S&OP at the executive level. J. Bus. Forecast. 2010; 29(4): 21–28.

[47] Yin RK. Case Study Research: Design and Methods. 4th ed. Sage Publications, Thousand Oaks, CA, 2009.

[48] Choi TY, Hong Y. Unveiling the structure of supply networks: case studies in Honda, Acura and Daimler Chrysler. JOM. 2002; 20(5): 469–493.

[49] Voss C, Tsikriktsis N, Frohlich M. Case research in operations management. Int. J. Oper. Prod. Manag. 2002; 22: 195–219.

[50] Eisenhardt KM. Building theories from case study research. AMR. 1989; 14(4): 532–550.

[51] Dyer WG, Wilkins AL. Better stories, not better constructs, to generate better theory: a rejoinder to Eisenhardt. AMR. 1991; 16(3): 613–619.

[52] Manuj I, Sahin F. A model of supply chain and supply chain decision-making complexity. Int. J. Phys. Distrib. Logist. Manag. 2011; 41(5): 511–549.

[53] Campbell DT. Pattern matching as an essential in distal knowing. in Hammond KR (ed.). The Psychology of Egon Brunsvik, New York: Holt, Rinehart &Winston, 1966; 81–106.

[54] Yin RK. Discovering the future of the case study. Method in evaluation research. Evaluation practice. 1994; 15(3): 283–290.

[55] Almutairi AF, Gardner GE, McCarthy A. Practical guidance for the use of a pattern-matching technique in case-study research: A case presentation. Nurs Health Sci. 2014; 16(2): 239–244. doi: 10.1111/nhs.12096 24251820

[56] Thomas A, Byard P, Francis M, Fisher R, White GRT. Profiling the resiliency and sustainability of UK manufacturing companies. J. Manuf. Technol. Manag. 2016; 27(1): 82–99.

[57] Ghobakhloo M. The future of manufacturing industry: a strategic roadmap toward Industry 4.0. J. Manuf. Technol. Manag. 2018; 29(6): 910–936.

[58] Zutshi A, Sohal AS. Integrated management system: the experiences of three Australian organisations. J. Manuf. Technol. Manag. 2005; 16(2): 211–232

[59] Burgess K, Singh PJ, Koroglu R. Supply chain management: a structured literature review and implications for future research. International journal of operations & production Management. 2006.

[60] Spina G, Caniato F, Luzzini D, Ronchi S. Assessing the use of external grand theories in purchasing and supply management research. Journal of Purchasing and Supply Management, 2016; 22(1): 18–30.

[61] Merton RK, Merton RC. Social theory and social structure. Simon and Schuster. 1968.

[62] Chicksand D, Watson G, Walker H., Radnor Z, Johnston R. Theoretical perspectives in purchasing and supply chain management: an analysis of the literature. Supply Chain Management: An International Journal. 2012; 7(4): 454–472.

[63] Williamsom OE. Transaction-cost economics: The governance of contractual relations. Journal of Economic Issues. 1979; 22: 233–261, 1979.

[64] Hackman JR, Wageman R, Ruddy TM, Ray C.R. Team effectiveness in theory and practice. Industrial and Organizational Psychology: Theory and practice, Oxford, UK: Blackwell. 2000.

[65] Tajfel H. Social psychology of intergroup relations. Annual Review of Psychology. 1982; 33(1): 1–39.

[66] Tajfel H, Turner JC. The social identity theory of intergroup behavior. In Worchel S., & Austin W. G. (Eds.). Psychology of intergroup relations. (2^nd^ ed.). Chicago, IL: Nelson-Hall. 1986; 7–24.

[67] Lange M, Mendling J, Recker J. An empirical analysis of the factors and measures of enterprise architecture management success, European Journal of Information Systems. 2016; 25(5): 411–431.

