# Supplementary material for: Global sales and operations planning: A multinational manufacturing company perspective
Source: PLoS One. 2021 Sep 21;16(9):e0257572. doi: 10.1371/journal.pone.0257572 (PMC8454961; doi:10.1371/journal.pone.0257572)
Supplement: S1 Appendix — (DOCX) [file pone.0257572.s001.docx]

**S1 Appendix**

The questionnaire utilized in the interviews is as follows.

Questionnaire:

1. Openings: introduction of the interviewer and the interviewee; overview of the research scope; assurance of confidentiality.
2. Demographic data: title and job history of participants; background of the organization.
3. Questions for executive directors:

- Do executives consider their S&OP process implementation strategic? Why?
- Does the organization ensure that the S&OP cycle meetings happen every month?
- Does the organization ensure attendance in the S&OP cycle meetings?
- Are reports showing S&OP metrics reviewed regularly?
- Are there formal Executive Meeting every month with defined agenda/outcomes, meeting minutes and defined participants? Do all participants attend the meetings?
- Are action items and decisions from Executive Meeting documented and tracked?
- Are pending issues from Pre-meeting resolved during the Executive Meeting?

1. Questions for managers and operational staff (functional areas):

- Does the organization ensure that the S&OP cycle meetings happen each month?
- Does the organization have an official list of empowered participants to support S&OP?
- Does the organization ensure attendance in the S&OP cycle meetings?
- Are reports showing S&OP metrics reviewed regularly?
- Is Data Gathering (S&OP Step one) fully completed prior to Demand Planning (Step two)?
- Does the organization generate a new forecast every month during S&OP cycle?
- Does the organization review excess and obsolete inventory, supplier lead times, SKU rationalization, forecast accuracy, customer service indicators, supply urgencies (e.g., air freights due to lack of product) during the S&OP cycle meetings?
- Does the organization have a formal Demand Planning Meeting with defined agenda/outcomes, meeting minutes and defined participants every month?
- Is Demand Planning (S&OP Step two) finalized before Supply Planning (Step three)?
- Is demand plan compared with budget and are financial gaps determined?
- Does marketing provide new product information and promotions plans?
- Does sales provide information on price increases, seasonality, new customers and other initiatives that may impact the S&OP plan?
- Is finance involved and committed with the S&OP process? What is its role?
- Does the organization have a formal Supply Meeting with defined agenda/targets/outcomes, meeting minutes and defined participants?
- Is Supply Planning (S&OP Step three) 100% finalized before Pre-meeting (Step four)?
- Are capacity and supply constraints identified, documented, addressed and regularly reviewed during Supply Planning?
- Does the organization have a formal Pre-meeting with defined agenda/ outcomes, meeting minutes and defined participants every month?
- Does the scope of Pre-meeting include finding solutions for issues related to Demand Plan x Supply Plan gaps? Is it documented and communicated to appropriate people?
- Is Pre-meeting (S&OP Step four) 100% completed before the Executive Meeting?
- Is an agenda for the Executive Meeting (Step five) prepared in the Pre-meeting (Step four)?
- What metrics are calculated, shared, and reviewed during the S&OP cycle? Are there defined targets for those metrics?
- Can the S&OP manual and documents regarding the S&OP process be seen?
- How is the global Roll-up step being implemented (Step six)? What are the main challenges observed? / How the Global executive meeting has being implemented (Step seven)?

1. Additional unplanned / floating points:

- Could you tell me more about that? / explain in more details? / provide more examples?
