# Supplementary material for: Global sales and operations planning: A multinational manufacturing company perspective
Source: PLoS One. 2021 Sep 21;16(9):e0257572. doi: 10.1371/journal.pone.0257572 (PMC8454961; doi:10.1371/journal.pone.0257572)
Supplement: S2 Appendix — (DOCX) [file pone.0257572.s002.docx]

**S2 Appendix**

The observational guidelines utilized in the interviews are as follows.

**Table 1. Observational Guideline**

| **Cycle-step** | **Action** | **Main inputs** | **Participants** | **Regularity** | **Outputs** | **Metrics** |
| --- | --- | --- | --- | --- | --- | --- |
| Steps one to five in Mexico and Brazil and steps six and seven in the headquarter (U.S.) | What is done within each step? | Which data is used and when? What are the data sources, including information system? Who is responsible? | Who attends the meetings? What are their role, commitment, empowerment and frequency attending? | When do the meetings occur and with which regularity? Are there event driven meetings? | What are the outcomes (e.g., information, S&OP plan, and time horizon)? To whom are results sent? What are the inputs for the following step? Is there an agenda for the next step? | Are there any metrics associated to the step? Which? How are they used, by whom and with what objective? |
